# DT-YOLO: An Improved Object Detection Algorithm for Key Components of Aircraft and Staff in Airport Scenes Based on YOLOv5

**DOI:** 10.3390/s25061705

**Published:** 2025-03-10

**Authors:** Zhige He, Yuanqing He, Yang Lv

**Affiliations:** 1 School of Computer Science, Civil Aviation Flight University of China, Guanghan 618307, China; hezhige@cafuc.edu.cn; 2 College Office, Civil Aviation Flight University of China, Guanghan 618307, China; lvyang19950206@163.com

**Keywords:** airport apron, YOLOv5, transformer, dropout, deformable convolution, GIoU

## Abstract

With the rapid development and increasing demands of civil aviation, the accurate detection of key aircraft components and staff on airport aprons is of great significance for ensuring the safety of flights and improving the operational efficiency of airports. However, the existing detection models for airport aprons are relatively scarce, and their accuracy is insufficient. Based on YOLOv5, we propose an improved object detection algorithm, called DT-YOLO, to address these issues. We first built a dataset called AAD-dataset for airport apron scenes by randomly sampling and capturing surveillance videos taken from the real world to support our research. We then introduced a novel module named D-CTR in the backbone, which integrates the global feature extraction capability of Transformers with the limited receptive field of convolutional neural networks (CNNs) to enhance the feature representation ability and overall performance. A dropout layer was introduced to reduce redundant and noisy features, prevent overfitting, and improve the model’s generalization ability. In addition, we utilized deformable convolutions in CNNs to extract features from multi-scale and deformed objects, further enhancing the model’s adaptability and detection accuracy. In terms of loss function design, we modified GIoULoss to address its discontinuities and instability in certain scenes, which effectively mitigated gradient explosion and improved the stability of the model. Finally, experiments were conducted on the self-built AAD-dataset. The results demonstrated that DT-YOLO significantly improved the mean average precision (mAP). Specifically, the mAP increased by 2.6 on the AAD-dataset; moreover, other metrics also showed a certain degree of improvement, including detection speed, AP50, AP75, and so on, which comprehensively proves that DT-YOLO can be applied for real-time object detection in airport aprons, ensuring the safe operation of aircraft and efficient management of airports.

## 1. Introduction

The flourishing development of the aviation industry has led to swift growth in the number of airports and flights, making it more and more convenient for people to travel by air. The safe and efficient operation of aircraft has become one of the most important considerations in the aviation industry. In order to ensure the safety and efficiency of flights, especially regarding airport aprons, it is particularly important to be able to detect staff and the key components of aircraft accurately. However, there is currently a lack of models for the detection of staff and aircraft key components in airport aprons; such detection is generally performed through manual visual inspection, which is inefficient and easily influenced by subjective factors, potentially leading to some objects being overlooked. The existing detection models generally struggle with low accuracy, the inability to detect in real-time, and difficulties regarding deployment, which can lead to airport accidents. On 2 January 2024, Japan Airlines Flight 516 collided with a fixed-wing aircraft of the Japan Coast Guard Agency on the runway after landing at Haneda Airport and caught fire. This resulted in 5 fatalities and 15 injuries, along with serious consequences in the form of the cancellation of over 1450 flights and a 70% reduction in the overall operational efficiency of the airport [1]. On 6 April 2024, two aircraft (British Airways and Virgin Atlantic) collided at Terminal 3 of London Heathrow Airport. Although no casualties or injuries occurred, both planes sustained significant damage, resulting in economic losses [2]. On 6 February 2024, a staff member accidentally fell out of a vehicle while on duty near an aircraft at Hong Kong International Airport, leading to an immediate fatality [3]. According to statistics, the proportion of incidents between 2005 and 2023 during takeoff and landing was as high as 67%, 53% of which occurred during landing (among all aviation accidents). The incidents referred to above fully demonstrate that the inability to accurately and quickly identify airport objects severely impacts flight safety and airport operational efficiency. Therefore, there is an urgent need for a model that can quickly and accurately identify objects in airports. However, there are still many challenges in object detection for airport aprons, such as the following:

- For the images collected from airports, some objects may become distorted or deformed due to factors such as the angles of the camera and the shooting environment, which increase the difficulty of detection;
- The traditional models for object detection are restricted by the receptive field, making it difficult to capture global information and dependency images, which limits the feature representation and affects the performance of models;
- For the scenario of airport aprons, existing models typically struggle with issues such as low accuracy, slow speed, and lack of real-time performance, thus failing to meet the increasingly complex task requirements and operational modes of modern airports.

With the introduction and development of the Smart Civil Aviation concept, modern information technology has become one of the key means to improve operational efficiency, ensure safety, and optimize service quality in the civil aviation industry; its core goal is to enhance the automation and intelligence level of airports by using advanced technologies [4]. Computer vision based on deep learning has provided new technical paths and ideas for the rapid and accurate identification of objects in airport apron scenes. As one of the core tasks in computer vision, object detection focuses on recognizing the categories of objects and positioning them in images; object detection has attracted widespread attention in both academia and industry and has been widely applied in multiple fields, such as autonomous driving [5], medical imaging [6], industrial production [7], security monitoring [8], and so on. In addition, the introduction of deep learning has enabled object detection models to achieve superior performance and adaptability, allowing them to handle various complex scenes. Among the numerous target detection algorithms, YOLOv5 is widely used in various fields due to its high detection accuracy and fast detection speed, which can meet the real-time detection requirements of airport aprons. In addition, the existing research results have fully proven its stability and progressiveness. Therefore, we use the popular object detection framework YOLOv5 as a baseline and improve upon it in this study; this enabled it to handle complex and varied object detection tasks in airport apron scenes, ensuring high speed while maintaining high accuracy. The improved YOLOv5 can effectively enhance airport security management and monitoring efficiency, providing strong technical support for the development of Smart Civil Aviation.

The following are the main contributions of this paper:

1. We constructed a dataset called AAD-dataset for airport apron scenes based on real-world surveillance video collected in airports, including 8643 images, 85,275 instance targets, and six categories;
2. We developed the D-CTR module, which integrates the Transformer self-attention mechanism with a dropout layer to overcome the limitations of traditional convolutional neural network receptive fields. This enhances feature representation while preventing model overfitting and improves generalization;
3. We used deformable convolution to extract features from images, which enhances the ability of the model to detect deformable objects while improving its adaptability to objects with different scales, thus further enhancing detection capacity;
4. We improved the loss function to address the discontinuity and instability issues of GIoULoss, which can lead to sudden changes in loss values and gradient explosion in certain situations; this modification further enhances the stability and accuracy of model training.

The rest of this paper is organized as follows. We introduce some related works in Section 2. Section 3 introduces the self-built dataset called AAD-dataset. Section 4 introduces the overall structure of DT-YOLO and provides detailed improvements for each component. In Section 5, we conduct experiments on AAD-dataset. Finally, we give our conclusions in Section 6, pointing out some existing problems and prospects for future work.

## 2. Related Works

In this section, we briefly introduce some related work, including object detection algorithms and their utilization in the aviation industry.

### 2.1. Algorithms for Object Detection

Object detection aims to classify specific objects and locate their positions in an image. The early object detection algorithms relied mainly on manually designed feature extractors and classifiers to classify and locate objects in images. For example, AdaBoost classifiers based on Haar features [9] use the approach of step-by-step weighted voting to detect objects; this was one of the representative algorithms for early face detection. DPM (deformable part model) [10] combines HOG features and the SVM classifier for object detection, making it one of the top traditional object detection algorithms. From a historical perspective, traditional object detection algorithms played an important role in early detection tasks but relied on manual features; this limited their expression, meaning that they could only be applied to some specific tasks, leading to weak generalization ability. Additionally, due to the constraints of classifier performance, the traditional object detection methods struggled with handling scenes with multi-scale and multi-pose objects. In summary, the traditional object methods are no longer sufficient for modern and increasingly complex object detection tasks.

At present, with the development of AI, the mainstream object detection algorithms have gradually shifted from traditional methods to deep learning-based algorithms, which can automatically extract features from images by using CNNs; they perform classification and regression to classify and locate specific objects, improving the speed and accuracy of object detection significantly. Based on deep learning, there are two object detection frameworks: one-stage and two-stage.

The two-stage version splits a detection task into two phases; first, it generates the candidate regions and then performs classification and regression on those regions. There are many different algorithms, such as [11], Fast R-CNN [12], Faster-RCNN [13], Mask-RCNN [14], Libra R-CNN [15], Cascade R-CNN [16], and so on [17,18].

The one-stage object detection algorithms directly predict the category and bounding box of objects in one step, resulting in a significant improvement in speed, and are widely used in real-time tasks and resource-constrained scenarios [19]. The representative version is You Only Look Once (YOLO), which can directly perform both object classification and localization within a single neural network by transforming object detection into a regression problem, significantly boosting detection speed. Accuracy has also been progressively enhanced with continuous iterations of and improvements to YOLO, making it one of the most important object detection algorithms. J. Redmon [20] proposed the YOLOv1 object detection algorithm, which can divide an input image into several small grids and then extract features from each grid using convolutional and pooling operations to predict the bounding boxes and their categories with confidence; however, YOLOv1 performed poorly in the detection of small and overlapping objects due to limitations in the number of predicted boxes and handled classes. J. Redmon made some improvements to YOLOv1 and proposed YOLO9000 [21], which included additional batch normalization after convolution layer operation to enhance generalization ability. The Darknet-19 network was introduced to strengthen feature extraction, adopting an anchor box. However, the deeper network structure also increased model complexity and training difficulty. J. Redmon [22] proposed YOLOv3 with Darknet-53 as the backbone, which improved the capability of detecting small objects by making predictions within multiple layers to detect multi-scale objects; enhancements to the classifier and loss function were made to better detect multiple objects. A. Bochkovskiy [23] introduced YOLOv4, which incorporated data augmentation to further improve feature extraction efficiency and model performance. Jocher, G [24] proposed YOLOv5, with a lightweight design offering higher flexibility while ensuring high detection speed and accuracy, making it suitable for multi-scene deployment; as such, it has become the most popular version in applications. The subsequent YOLO versions, such as YOLOv6 [25], YOLOv7 [26], YOLOv8 [27], and so on [28], have further improved upon the previous algorithms [29].

In recent years, many researchers have proposed new object detection approaches, such as RSUD20K [30], which is a new dataset for understanding road scenes. It contains over 20 K high-resolution images (from the perspective of driving) on roads in Bangladesh and includes 130 K bounding box annotations for 13 objects. The dataset not only serves as a benchmark for addressing existing vision challenges but also provides a platform for developing algorithms tailored for autonomous driving that can understand road scenes.

One-stage object detection algorithms can directly extract features from images without generating candidate regions, which significantly improves the speed of detection compared to two-stage object detection algorithms. In addition, accuracy has also been significantly improved through the iteration of YOLO versions. Therefore, we used YOLOv5 as the baseline and made some improvements to it to better adapt it to airport apron scenes.

### 2.2. The Utilization of Computer Vision in the Field of Aviation

The utilization of computer vision in the civil aviation industry has gradually become an important technical support for aviation safety, fault detection, aircraft maintenance, and so on. Anurag Upadhyay [31] proposed U-Net for the detection of engine blade defects based on deep learning, which addresses the issues of data imbalance and small objects by testing different loss functions and combining a customized GAN model; image sharpening and denoising are applied to cope with the motion blur caused by the high-speed movement of engine blades. Zejiang Shen [32] used a fully convolutional network to identify and locate cracks and burns—the two main types of damage—in endoscopic images, addressing the issue of previous methods struggling with the complex and noisy internal scenes of aircraft pipelines. Yi Qu [33] made improvements to YOLOv5 by replacing the detection module, introducing NWD detection metrics, and adding sampling operators, which improved the average accuracy of detection and facilitated the lightweight deployment of the model. Runyuan Wen [34] proposed LESM-YOLO by adding a lighting enhancement module and introducing mixed local channel attention for defect detection in aircraft conduits; this improved the accuracy and robustness under low-light conditions and made it easier to deploy due to the use of deep convolution to reduce the parameter count. L. Connolly [35] applied YOLOv8 for visual inspections of aircraft, optimizing the model’s detection performance through various hyperparameter combinations to successfully detect surface defects on aircraft.

On the other hand, computer vision technology has been widely applied in various aspects of intelligent airports, such as X-ray contraband detection [36]. Chunjie Ma [37] proposed EAOD-Net, introducing Gabor convolution layers, deformable convolution, and spatial attention mechanisms for feature enhancement; dense local regression and ROI pooling for classification and regression were also used, making the X-ray contraband detection results more accurate. Qianxiang Cheng [38] proposed X-YOLO for contraband detection, which combines feature fusion and attention mechanisms and integrates an improved dynamic head module, achieving impressive results on both the HiXray and OPIXray datasets. In the area of airport remote sensing, Ting Wang [39] constructed a dataset for small-object aircraft and proposed a circular frequency filtering detection algorithm. This was integrated with a mean-shift clustering algorithm to enhance the performance of small-object aircraft detection. Lijian Yu [40] proposed the YOLO-FRS network architecture for detecting aircraft in remote sensing images, which used feature-response-separating deformable convolution modules and added semantic segmentation supervision based on stacked DCNs. Soft label annotations and loss calculation methods were also applied, demonstrating good performance on their self-built dataset. Qiang Wang [41] proposed the TPH-YOLOv5-Air remote sensing airport detection network based on adaptive spatial feature fusion with an adaptive parameter adjustment module; this improved feature extraction efficiency and size invariance, enhancing remote sensing-based airport detection performance. We summarize the literature mentioned above in Table 1.

However, there have been relatively few object detection models for airport apron scenes due to the lack of datasets and the complexity of the airport environment. Zonglei Lyu [42] reduced the parameter count and computational complexity of a model using lightweight techniques, which enabled its deployment on edge cloud systems with limited computing power for real-time monitoring of airport apron videos; however, the model can only detect entire aircraft and is unable to perform more detailed detection of key components of an aircraft. Jun Li [43] proposed NCABLD, a three-module network with a progression from coarse-grained to fine-grained detection. However, it can only recognize the staff and their actions, and its performance is lacking in environments with occluded objects or poor lighting. Therefore, we designed a novel object detection architecture based on YOLOv5 for airport apron scenes, named DT-YOLOv5, in order to enhance the recognition ability of airport apron objects and improve airport safety and operational efficiency.

## 3. AAD-Dataset

In this section, we provide a detailed introduction of the ADD-dataset that was constructed in this study, including the data collection, data annotation, and analysis of the instances contained in the dataset.

### 3.1. The Collection of AAD-Dataset

**Figure 1:**
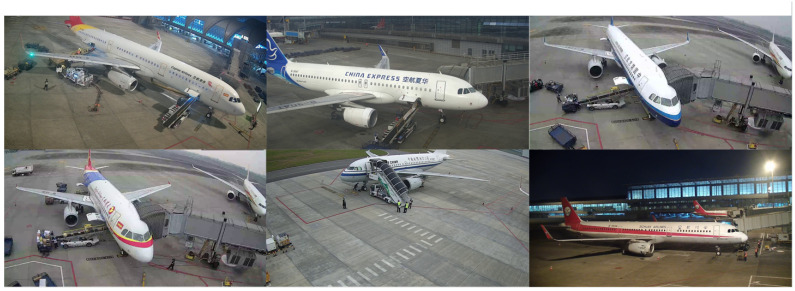
Part of the dataset.

At present, there is a lack of datasets for airport apron scenes; even though the existing open-source datasets contain objects such as aircraft and staff, their number and scale are quite small, making it difficult to apply them to complex airport apron scenes. Therefore, we constructed an image dataset named AAD-dataset by randomly sampling 90 real surveillance videos from Shuangliu International Airport and Tianfu International Airport, which contain a large number of complex airport apron scenes. The quality of the images in the dataset is the same and includes multiple environments, such as rainy, night-time, and so on. Part of the dataset is shown in Figure 1.

The AAD-dataset constitutes a total collection of 8643 high-quality images with 80,275 instances after the removal of background interference and poor-quality blurry images. Then, we divided the whole dataset into the training, testing, and validation sets at a ratio of 3:1:1, which sees 5187, 1728, and 1728 images, respectively. The resolutions of the images are either 1920 × 1080 or 1280 × 720 pixels, due to the limitations of the surveillance cameras. Our dataset contains images captured at different moments with significant variations in lighting conditions, shooting angles, object orientations, and so on. The diversified images provide richer feature representation capabilities for the model, which is particularly suitable for the object detection task in complex and variable airport apron scenes. Additionally, the robustness and accuracy of the model can be effectively improved in changing environments by using these carefully selected and annotated images.

### 3.2. The Annotation of the AAD-Dataset

**Figure 2:**
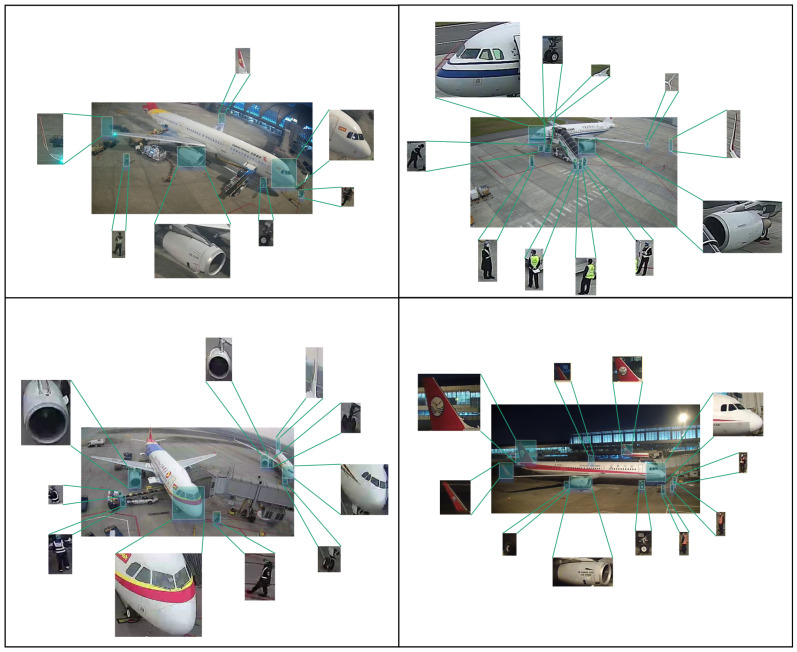
Annotation of some of the samples in the training dataset.

We utilized labeling to annotate the bounding boxes for all objects in the training set in the standard format of $\left(class,x_{center},y_{center},width,height\right)$; this provided high-quality annotated images for the training of the model, where “class” represents the category of the object within the bounding box, $x_{center}$ and $y_{center}$ represent the center coordinates of the bounding box, respectively, and width and height denote the width and height of the bounding box. The utilization of labeling for annotation allows the position, size, and category of each object in the image to be accurately recorded, allowing the model to learn the specific features and spatial distribution of the objects during training. Figure 2 illustrates the annotation of some samples from the dataset, providing a visual understanding of the annotation and the accuracy of the requirements, which lays a solid foundation for subsequent tasks of object detection.

### 3.3. The Categories of AAD-Dataset

In this study, we detected the key components of aircraft and staff within airport apron scenes; in particular, the key components of the aircraft include the tail, nose, wings, engines, and landing gear. Therefore, we aimed to classify these six types of targets, ensuring that the model can effectively recognize and locate different types of targets. According to the annotations in the dataset, the tail category contains 10,386 instances, the nose category contains 10,379 instances, the wings category contains 18,986 instances, the engine category contains 11,814 instances, the landing gear category contains 8578 instances, and the personnel category contains 31,946 instances; the total number of instances across all categories amounts to 80,275. This substantial number of instances fully reflects the diversity and complexity of the objects in airport apron scenes, where the spatial distribution, scale, and orientation of targets exhibit significant variations. The large-scale and rich instances in AAD-dataset provide a solid data foundation for this research.

### 3.4. Analysis of Category Scales in the AAD-Dataset

For the AAD-dataset, we measured the size of each object based on the number of pixels within its bounding box, and we divided all the objects into three types: small, medium, and large. The small-sized objects comprise the largest proportion, accounting for 51.8% of the total instances, primarily including the staff, landing gear, and wings, frequently appearing in airport apron scenes. The medium-sized objects account for 36.6% of the instances, including engines and tails, which play a crucial role in airport apron scenes, making their detection moderately challenging. The large-sized objects, which comprise the smallest proportion at 11.6%, mainly include the aircraft’s nose, tail, and engines. They are more prominent in the image but still pose certain challenges in the detection process due to their rarity. Table 2 summarizes the details of the number of instances and proportions for each category, providing a clear reference for subsequent object detection and model evaluation tasks.

### 3.5. Analysis of the Object Orientation in AAD-Dataset

**Figure 3:**
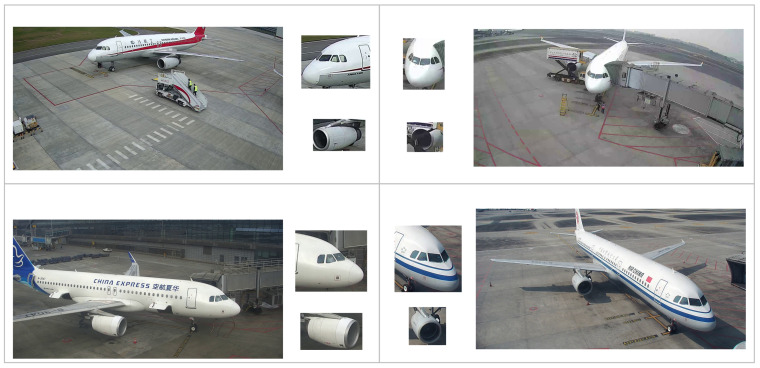
Objects with different orientations in the AAD-dataset.

The images in AAD-dataset are derived from videos captured by airport surveillance cameras. Due to differences in camera installation angles, distances, and other environmental factors, objects appear at different orientations across the images. Especially in dynamic and complex airport apron scenes, the objects’ posture and direction in the surveillance videos can change continuously. The variations significantly increase the complexity and difficulty of detection, which means that the object detection models must be able to handle the objects’ orientation changes and irregular spatial distributions. Figure 3 illustrates some objects with varying orientations.

## 4. Methodology

In this section, we first give a brief overview of DT-YOLO and then introduce the various components of DT-YOLO in detail, including the construction of D-CTR and the process of deformable convolution. Finally, we demonstrate the improvements to the loss function.

### 4.1. Overview of DT-YOLO

**Figure 4:**
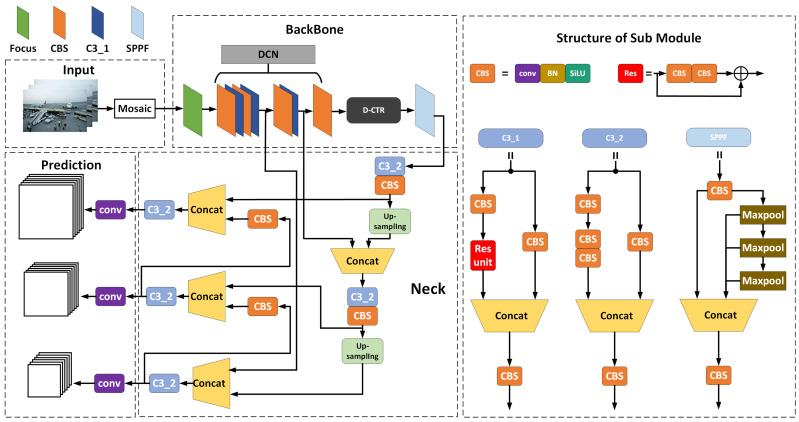
The overall architecture of DT-YOLO consists mainly of four parts, including the Input layer, Backbone, Neck, and Prediction. Three different size sizes of feature maps are output and used for different scale object detection.

End-to-end architecture-based object detection algorithms have gained widespread attention in both industry and academia due to their fast detection speed and excellent detection performance. It is essential to establish object detection models with higher accuracy, faster detection speeds, and better performance for airport apron scenes with a high volume of aircraft and numerous staff; this is expected to help meet the demands of the detection for aircraft key components and personnel in apron scenes. Therefore, we propose DT-YOLO, the architecture of which is shown in Figure 4.

In the input layer, DT-YOLO first employs the technique of Mosaic data augmentation for image prepossessing, which combines four images with different semantics into a new image; then, it introduces multiple objects into the same scene to simulate the complexity of real-world environments by changing the visual features. The Mosaic not only increases the diversity of the dataset but also enhances the robustness of the model, improving its ability to detect objects of different sizes. Additionally, the input layer ensures the image’s consistency through resizing and normalization and enhances the diversity by introducing operations like random rotation and flipping.

The backbone contains modules such as Focus, CBS, C3_1, and SSPF, and we introduce the D-CTR module. The Focus module is the first module of the backbone, which is used to reorganize the pixel information of the original image. It generates four images by slicing the original image and then concatenates them; this expands the channel information by four times, thus enhancing the feature representation capability and facilitating feature extraction and fusion in the subsequent structures. The CBS module consists of CONV (convolution), BN (batch normalization), and Sliu, which is the standard convolution module. CONV is responsible for extracting features from images, while BN performs normalization on the images; finally, Sliu introduces non-linear features. The C3_1 module, based on the CSPnet architecture, contains the ResUnit and CBS modules. The images input into C1_3 are processed in two parallel paths: one path directly enters CBS for feature extraction, and the other moves through the CBS and residual blocks for feature extraction; then, the features from these two paths will be concatenated, obtaining the feature map, which will be input into CBS to output the final results. In the SSPF module, the pooling operations are performed on the images by using different-sized pooling kernels, enabling the model to capture features of objects with different sizes. It then concatenates these feature maps to integrate features from various scales to form a new feature map. We constructed the D-CTR module based on the CTR3 module, which is located between the last CBS module and the SPPF module in the backbone. It can process the feature maps after being processed by the backbone. The D-CTR module incorporates the Transformer self-attention mechanism [44,45] and Dropout layer [46], which can enhance the feature representation and semantic information, enabling self-attention for features and cross-channel information interaction.

The Neck part adopts FPN and PAN structures, including the CBS and C3_2 modules. The CBS module comprises CONV, BN, and Sliu, which is the same as the backbone. The C3_2 module, based on the CSPnet architecture, is derived by setting the shortcut to False in the ResUnit of C3_2, and its processing flow to images is similar to C3_1. The Neck part effectively integrates deep semantic information with shallow detail information through upsampling and feature concatenation. In addition, it enhances the richness and hierarchical structure of the features by facilitating bidirectional feature flow, thus optimizing detection performance.

The Prediction part is responsible for the precise classification and location of objects, which uses SoftMax for classification and performs bounding box regression to accurately locate the objects.

### 4.2. The D-CTR Module

**Figure 5:**
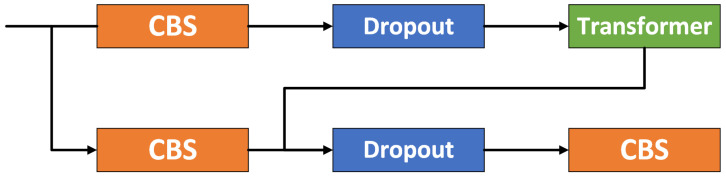
The structure of D-CTR.

Traditional CNNs are limited by their receptive field, which typically allows them to extract only local features from the image and makes it difficult to handle long-range dependencies in images. Increasing the number of CNN layers can expand the receptive field, to some extent, but this also brings more additional computational overhead. To address this issue, we modified the traditional CTR3 module and incorporated the Transformer to expand the receptive field. This allows it to handle objects with complex semantic information or fine details, thus enhancing the capability of DT-YOLO to capture global information from images. Additionally, we also introduced a Dropout layer, which randomly “drops” some neurons in the neural network during training to prevent overfitting caused by unnecessary or noisy features, which could degrade model performance, thus reducing the risk of overfitting and improving the model’s robustness. The D-CTR structure is shown in Figure 5.

#### 4.2.1. The Transformer Self-Attention Mechanism

**Figure 6:**
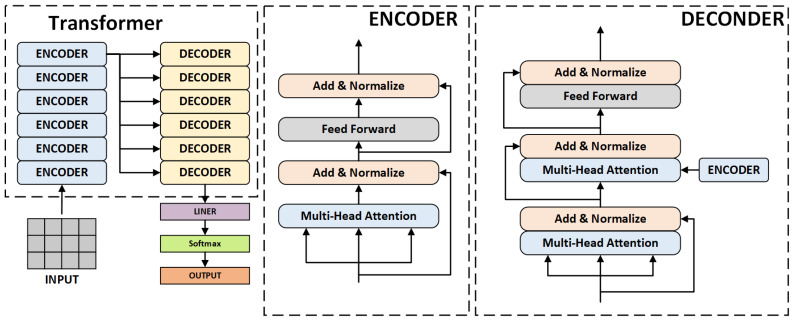
The overall architecture of Transformer.

The emergence of Transformer has significantly changed the mode of processing sequential data. Unlike traditional CNNs, Transformer can capture the long-range dependencies within the input sequence data through the use of a self-attention mechanism. It consists of six encoder layers and six decoder layers, where each encoder layer contains the muti-head attention mechanism, feed-forward neural network, normalization layer, and residual links, which are responsible for encoding the input sequential data into context representations and mapping them to a fixed-dimensional representation space, allowing the model to better capture the relationships between different positions within the input sequence. Each decoder is similar to the encoder, and each layer also includes an additional muti-head attention mechanism and normalization layer, which are used to extract information from the representation space and generate the object sequence. The architecture of Transformer is shown in Figure 6.

In the Transformer model, the encoded matrix output from the encoder is passed to the decoder, and the final results are obtained after linear processing and passing through the SoftMax layer. In order to achieve the parallel processing of sequences, the Transformer eliminates the recursive architecture of the RNN and adopts a self-attention mechanism to establish relationships between different positions within the input sequence. This enables the model to better extract contextual information and global features from the input images. For a certain input sequence, $X=\left[x_{1},x_{2},x_{3},\ldots ,x_{n}\right]$, the input $x_{i}\left(i\in \left[1,n\right]\right)$ in any position will generate *Q* (query), *K* (key) and *V* (value) through three different linear transformations, as shown in Equation (1).

(1) $$\begin{array}{c} Q=XW_{Q},K=XW_{K},V=XW_{V},\\ Q=[q_{1},q_{2},q_{3},\ldots ,q_{n}],\\ K=[k_{1},k_{2},k_{3},\ldots ,k_{n}],\\ V=[v_{1},v_{2},v_{3},\ldots ,v_{n}] \end{array}$$

In Equation (1), $W_{Q}$, $W_{K}$, and $W_{V}$ are the parameter matrices, which can be obtained through training predefined based on data features, and the value of *q*, *k*, and *v* for each position in the sequence can be derived. Each position in the input sequence needs to calculate its score regarding relevance with other positions in the sequence to determine its association with other positions in the model. First, the similarity, $\alpha_{ij}$, between position $x_{i}$ and other positions through the dot product should be calculated, as shown in Equation (2).

(2) $$\begin{array}{c} \alpha_{ij}=q_{i}k_{j},\\ A=\left(\begin{array}{cccccc} \alpha_{11} & \alpha_{12} & \cdots & \alpha_{1j} & \cdots & \alpha_{1n}\\ \alpha_{21} & \alpha_{22} & \cdots & \alpha_{2j} & \cdots & \alpha_{2n}\\ \vdots & \vdots & \ddots & \vdots & \cdots & \vdots \\ \alpha_{i1} & \alpha_{i2} & \cdots & \alpha_{ij} & \cdots & \alpha_{in}\\ \vdots & \vdots & \vdots & \vdots & \ddots & \vdots \\ \alpha_{m1} & \alpha_{m2} & \cdots & \alpha_{m4} & \cdots & a_{mn} \end{array}\right) \end{array}$$

In Equation (2), $\alpha_{ij}={q_{i}}^{T}k_{j}$, where *T* denotes the transpose. The larger the value of $\alpha_{ij}$, the greater the similarity between position *i* and *j*. The value of the dot product will be divided by $\sqrt{d_{k}}$ to prevent the gradient from disappearing or exploding, representing the key’s dimension for scaling. Then, the scaled similarity is normalized (using SoftMax) to the sum of 1, which is used to represent the weight of each position, as shown in Equation (3).

(3) $$\begin{array}{c} W\left(Q,K,V\right)=softmax\left(\frac{A}{\sqrt{d_{k}}}\right)\\ \frac{A}{\sqrt{d_{k}}}=\left(\begin{array}{cccccc} \frac{\alpha_{11}}{\sqrt{d_{k}}} & \frac{\alpha_{12}}{\sqrt{d_{k}}} & \cdots & \frac{\alpha_{1j}}{\sqrt{d_{k}}} & \cdots & \frac{\alpha_{1n}}{\sqrt{d_{k}}}\\ \frac{\alpha_{21}}{\sqrt{d_{k}}} & \frac{\alpha_{22}}{\sqrt{d_{k}}} & \cdots & \frac{\alpha_{2j}}{\sqrt{d_{k}}} & \cdots & \frac{\alpha_{2n}}{\sqrt{d_{k}}}\\ \vdots & \vdots & \ddots & \vdots & \cdots & \vdots \\ \frac{\alpha_{i1}}{\sqrt{d_{k}}} & \frac{\alpha_{i2}}{\sqrt{d_{k}}} & \cdots & \frac{\alpha_{ij}}{\sqrt{d_{k}}} & \cdots & \frac{\alpha_{in}}{\sqrt{d_{k}}}\\ \vdots & \vdots & \vdots & \vdots & \ddots & \vdots \\ \frac{\alpha_{m1}}{\sqrt{d_{k}}} & \frac{\alpha_{m2}}{\sqrt{d_{k}}} & \cdots & \frac{\alpha_{mj}}{\sqrt{d_{k}}} & \cdots & \frac{\alpha_{mn}}{\sqrt{d_{k}}} \end{array}\right) \end{array}$$

Based on Equation (3), the weights of the attention for each position in the input sequence were obtained. The higher weight indicates that the current position is more focused on another position, thus contributing more to the final results. Finally, the weight vector is used to perform a weighted sum with the value vectors, obtaining the output for each position, as shown in Equation (4).

(4) $$Attention\left(Q,K,V\right)=W\left(Q,K,V\right)\times V$$

$Attention\left(Q,K,V\right)=\left[y_{1},y_{2},y_{3},\ldots ,y_{n}\right]$ represents the output at each position in the sequence. Through this method, the output at each position can fully integrate semantic information from other positions, producing the optimal representation for each position.

To further enhance the expressiveness of the attention mechanism, Transformer introduces multi-head attention, which maps *Q*, *K*, and *V* to multiple subspaces with independent parameters, allowing the attention mechanism of each subspace to be computed in parallel, thus capturing contextual information from different perspectives. Finally, the attention from multiple heads is concatenated and linearly transformed, as shown in Equation (5).

(5) $$MultiHead\left(Q,K,V\right)=Concat\left(head_{1},head_{2},head_{3}\ldots ,head_{h}\right)\times W_{O}$$

**Figure 7:**
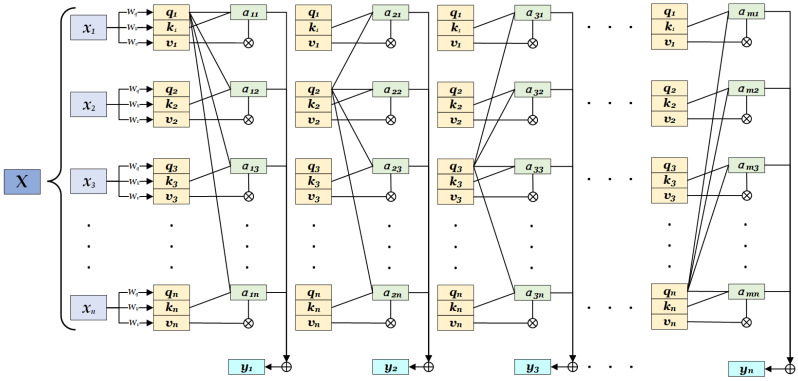
Data processing in the Transformer.

In Equation (5), $head_{i}=Attention\left(Q_{i},K_{i},V_{i}\right)$ represents the output of a certain head in the multi-head attention, which enables the model to learn multiple information from different subspaces, improving the performance of the model. The data processing in the Transformer is shown in Figure 7.

**Figure 8:**
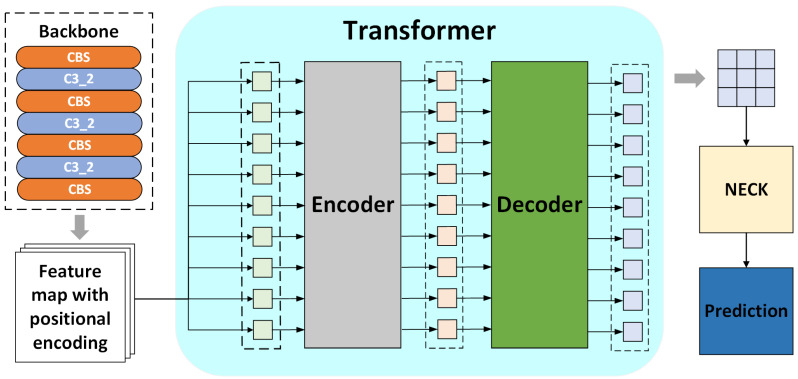
Image processioning in the Transformer.

After the images are handled by the backbone, they will be flattened into a series of feature vectors, each representing a region or pixel in the image. The feature vectors contain visual information about the image, such as color, texture, and shape. However, the Transformer does not have inherent sensitivity to the positional information of these regions, as it lacks the spatial constraints present in CNNs. Therefore, in order to enable the Transformer to understand and utilize position-related information, it is necessary to process the feature map output from the backbone to make it compatible with the Transformer’s input, providing each feature vector with unique positional information; this ensures that each feature vector carries not only visual information but also spatial information. The Transformer can understand the relative positional relationships of each pixel or region in the image using this method, thus effectively modeling the long-range dependencies. The processed feature map is then input into the Transformer encoders and decoders for further feature extraction and analysis. The overall processing flow of the images is shown in Figure 8.

As we can see in Figure 8, a multi-level feature map is obtained after the image undergoes feature extraction through the backbone. The feature map is then divided into nine regions with positional information, which are input into the Transformer; then, the relationships between regions with different features can be learned through a series of self-attention layers and be modeled, enabling the model to obtain the global contextual information of the feature map. This captures the long-range dependencies and outputs a feature map that contains global information.

The introduction of the Transformer self-attention mechanism breaks the locality bias inherent in traditional CNNs, making the receptive field of CNNs no longer limited to fixed local regions; this enables the model to capture long-range dependencies between different regions and deepen its understanding within global contextual information, thereby enhancing its ability to model distant objects. Especially in airport apron scenes, surveillance cameras can capture multiple similar distant objects in a single image, which may have semantic relationships. The Transformer can capture these to enhance the capability of the model to model these distant objects, thus improving the object detection accuracy and the overall performance of the model. Additionally, with the introduction of multi-head attention, the Transformer encoder can process multiple attention heads in parallel and adaptively adjust its focus according to the different attention heads, which allows it to capture information at multiple scales and dynamically fuse features; this significantly enhances the ability of the model to detect complex scenes of airport aprons and multi-scale objects.

#### 4.2.2. The Dropout Layer

The introduction of the Transformer significantly enhances the expressive capability of the model. Still, it may also cause the model to capture unnecessary or noisy features, increasing the risk of overfitting and degrading model performance, which could lead to excellent performance on the training set but poor generalization on the test set. In this study, we introduce the Dropout layer in the D-CTR module to enhance robustness to address this issue.

**Figure 9:**
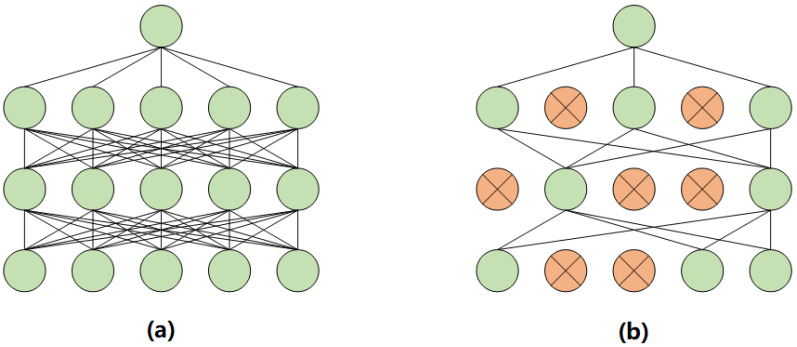
The structure of Dropout. (**a**) The typical fully connected neural network structure, and (**b**) the neural network structure after a random “drop out” of a portion of neurons, where the orange dots represent the discarded neurons.

Dropout is a widely used regularization technique in deep learning, and the core idea is to randomly “drop out” a portion of neurons during the training process (setting the output of those neurons to zero), which means the dropped-out neurons do not participate in the forward and backward propagation processes. This forces the neurons in the network to become more independent during training, thus preventing certain neurons from relying too heavily on information from others. As a result, the network must depend on different combinations of neurons to learn features in each iteration. Dropout has achieved remarkable results in various deep learning tasks and has become one of the standard regularization techniques in deep learning. The structure of Dropout is shown in Figure 9.

The formula for the typical fully connected neural network output, *Y*, is shown in Equation (6):

(6) Y=f(WX+b),

where *X* represents the input vector, and *b* represents the bias vector; *f* is the activation function, and *Y* is the output vector. First, the input vector, *X*, will be processed through a linear transformation. Then, the activation function, *f*, introduces non-linearity, enhancing the expressive capability of the neural network, which enables the neural network to learn and simulate complex functions. The Dropout operation can be represented by a mask vector, *M*, where each element is independently sampled from a Bernoulli distribution, as shown in Equation (7):

(7) $$\begin{array}{c} M=[m_{1},m_{2},m_{3},\ldots ,m_{i},\ldots ,m_{n}],\\ m_{i}∼Bernoulli\left(p\right) \end{array}$$

where *p* represents the probability of discarding the neuron, and 1−p represents the probability of retaining the neuron; the *i*-th neuron will “drop out” when $m_{i}$ is zero. The output formula is shown in Equation (8) after applying the Dropout operation.

(8) $$\begin{array}{c} Y_{train}=f\left(\left(W⊙M\right)X+b\right) \end{array}$$

where ⊙ represents the matrix dot, and $Y_{train}$ represents the output of the training. As *M* is randomly generated in each training session, the structure of the network is slightly different during each forward propagation. This allows the model to learn different feature combinations during each training session. In the testing phase, the Dropout operation is turned off to maintain the consistency and stability of the model, and all neurons in the neural network will participate in the calculation. Therefore, the output of each neuron during testing is multiplied by 1−p as a scaling factor to ensure the consistent desired value in the training and testing phases. The output, $Y_{test}$, is shown in Equation (9).

(9) $$\begin{array}{c} Y_{test}=\left(1-p\right)X \end{array}$$

Equation (9) ensures that the desired output value of the model remains unchanged while avoiding the noise introduced. Dropout introduces randomness to prevent the neural network from relying on any specific neuron activation pattern, and the neurons in the neural network are randomly dropped during each forward propagation, encouraging the remaining neurons to combine and learn the features from the input data; this forces the model to learn different subsets of features during each training session, thus enhancing its robustness. Furthermore, Dropout reduces the dependency between neurons, preventing overfitting to certain specific features. Additionally, the random dropping of neurons also makes the network sparser, reducing computational load and redundant features and, ultimately, improving the computational efficiency and training speed of the model.

### 4.3. Deformable Convolution

Traditional CNNs typically use fixed-size convolutional kernels to extract features from fixed regions in the images. However, the convolutional kernels with fixed geometric structures have certain limitations, such as fixed receptive fields, unsuitability to deformation, a lack of sensitivity to scale changes, and so on. This limits the expressive power of the model when handling images with complex deformations or changes in object position, size, or angle, reducing its robustness and affecting its performance. To address the limitations, Dai [47] proposed deformable convolutional kernels in 2017, which enables the kernel to learn spatial offset values by introducing spatial position variability. This expands the flexibility of the convolution and allows the convolutional kernel to adjust its shape based on the objects in the input images adaptively and extract features from different regions; this improves the ability of the model to adapt to complex deformations, scale changes, and angle variations, significantly enhancing the performance of the model on complex images.

**Figure 10:**
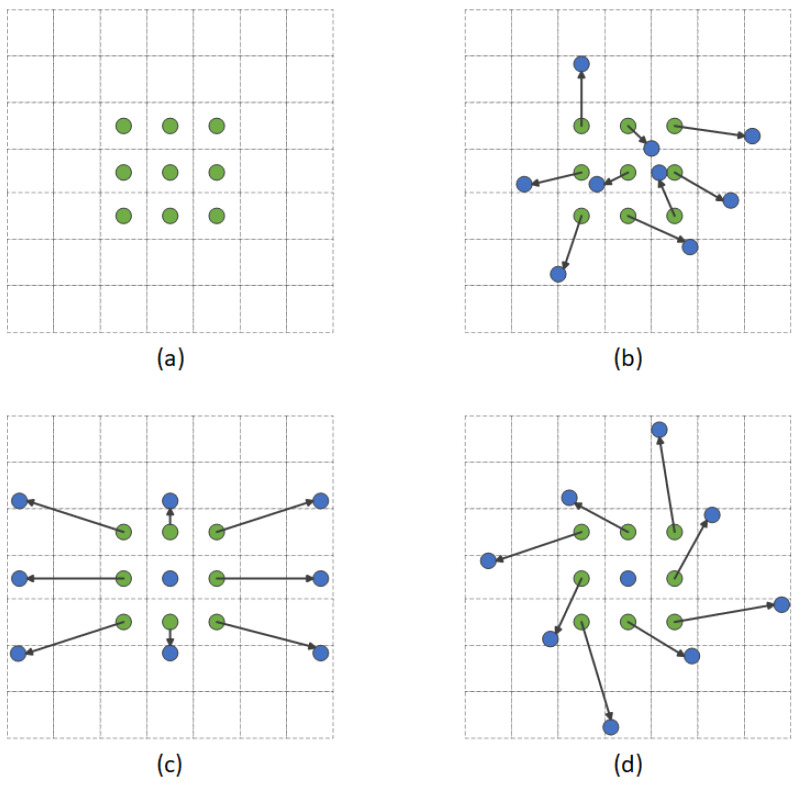
Comparison between traditional and deformable convolution. (**a**) Traditional convolution kernels. (**b**–**d**) Different processes of deformable convolutions. The features can be extracted at different positions around the sampling points after the introduction of offsets.

CNNs typically use a sliding convolutional kernel on the input image when traditional convolution is applied to extracting features. Each element of the kernel represents a weight, and the model performs a weighted sum with the corresponding region of the image when the kernel slides over the image, producing an output feature map, as shown in Equation (10):

(10) $$\begin{array}{c} Y\left(i,j\right)=X\cdot W\left(i,j\right)=\sum_{m}\sum_{n}X(i+m,j+n)W(m,n) \end{array}$$

where *X* represents the input image, *W* represents the weight of the convolutional kernel, and *Y* is the output feature map after the convolution operation. Traditional convolution can only extract features within a limited region due to its fixed geometric structure. Through introducing offsets, deformable convolution performs sampling operations around the sampling points, which gives the convolutional kernel a flexible structure and adjusts the sampling region dynamically, as shown in Figure 10.

In Figure 10, the green dots represent the original sampling points used in traditional convolution, the black arrows indicate the offsets added to the normal sampling points, and the blue dots represent the new sampling points. In detail, for a local region $x\left(i,j\right)$ in the input image and the convolutional kernel weights $W\left(m,n\right)$, the sampling position is changed by introducing the position offset $\Delta x\left(i,j\right)$, as shown in Equation (11):

(11) $$\begin{array}{c} Y\left(i,j\right)=X\cdot W\left(i,j\right)=\sum_{m}\sum_{n}X\left(i+m+\Delta x_{m},j+n+\Delta y_{m}\right)W\left(m,n\right) \end{array}$$

The input image first generates the offset for each point, which serves as the adjustment information for the new sampling points during the convolution operation. Specifically, for each position $\left(m,n\right)$ in the convolution kernel, the generated offsets $\Delta x_{m}$ and $\Delta y_{m}$ are used as the offsets of the convolution kernel, replacing the original sampling point $\left(i+m,j+n\right)$ with $\left(i+m+\Delta x_{m},j+n+\Delta y_{n}\right)$. The convolution kernel can adaptively perceive the content of the input image based on these offsets, and the weighted sum is performed at the offset positions.

The introduction of deformable convolution significantly enhances the model’s expressive power. In detail, the traditional convolution kernels are not flexible enough to adjust their receptive fields for deformed objects. Deformable convolution, which can dynamically adjust its receptive field through the learning of position offsets, is highly sensitive to the deformation of objects in the image; thus, it better adapts to various deformed objects and provides higher flexibility in handling complex scenes such as object deformation, rotation, and scale changes. Furthermore, deformable convolution allows the network to exhibit higher robustness, especially in images with complex object distributions; deformable convolution can dynamically adapt to these situations.

In the complex scenes of airport aprons, the objects in the images often exhibit deformations, occlusions, and scale differences, which increases the difficulty of target detection. For example, aircraft can appear at varying sizes depending on their distance, and staff may be partially occluded by other objects. In this situation, traditional CNNs may struggle to handle these variations, further reducing the accuracy of the model. By introducing deformable convolutions, the model can dynamically adjust the receptive field of the convolutional kernels, learning features that adapt to deformations and changes in the targets, which significantly enhances the ability of the model to detect objects, especially when handling issues such as deformation, occlusion, and scale differences.

### 4.4. The SLoss Function

In object detection tasks, the precision of object localization is crucial. However, the traditional IoU loss functions often fail to accurately measure the difference between the ground truth bounding box and the predicted bounding box when dealing with complex scenes and small objects. Rezatofighi et al. [48] proposed the GIoU loss function to address the shortcomings of traditional loss functions. By considering the intersection area and introducing the concept of the enclosing rectangle, effective loss values and more gradient signals can be provided when the two bounding boxes are far apart, avoiding the issue of gradient vanishing associated with IoU loss functions. The formula for GIoU is shown in Equation (12):

(12) $$\begin{array}{c} GIoU\left(B_{gt},B_{pred}\right)=IoU\left(B_{gt},B_{pred}\right)-\frac{C-(B_{gt}\cup B_{pred})}{C} \end{array}$$

where $B_{gt}$ represents the real box, $B_{pred}$ represents the predicted box, $IoU(B_{gt},B_{pred})$ represents the intersection and union ratio between the predicted box and the real box, and *C* represents the minimum area of the external rectangle with the two boxes. Equation (12) indicates that GIoU not only focuses on the intersection area between the two boxes but also on the gap between them; therefore, the GIoU can still measure the difference between the two boxes when the two boxes do not overlap, providing a smoother loss curve, which avoids the problem of sudden changes, making object positioning more accurate. The formula for $GIoU_{Loss}$ is shown in Equation (13):

(13) $$\begin{array}{c} GIoU_{Loss}=1-GIoU\left(B_{gt},B_{pred}\right) \end{array}$$

**Figure 11:**
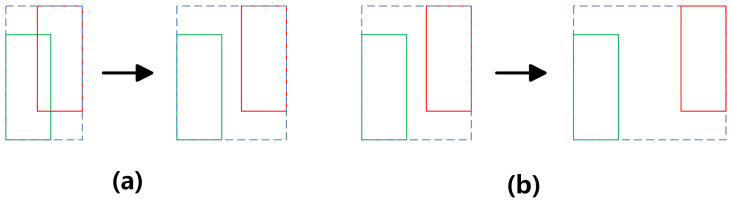
Discontinuity and instability. (**a**) Discontinuity; (**b**) instability.

$GIoU_{Loss}$ can achieve faster and more stable convergence when training the model, and it can be integrated into various object detection frameworks easily. However, it may cause discontinuity and instability when the predicted bounding box does not overlap with the ground truth bounding box at all. Here, discontinuity refers to the GIoU suddenly jumping from a non-zero value to −1, with the two boxes from the overlap becoming completely disjointed; in contrast, the optimization method, such as gradient descent, usually relies on the continuity and smoothness of the loss function to adjust the parameters of the model gradually. Discontinuity can cause the process of optimization to become unstable, resulting in lower model efficiency and performance. On the other hand, instability refers to extreme situations where a huge difference between the two boxes exists. For example, GIoU will consider the minimal enclosing area of the two bounding boxes when they do not overlap and the distance is large; the area of the minimal enclosing area may become extremely large in this situation, which could far exceed the union of the two boxes in the area. This causes the value of loss to increase sharply, and the sudden increase in the loss value generates large gradients, which may lead to the problem of gradient explosion during training, thus hindering the effective learning of the model. The situation of discontinuity and instability are shown in Figure 11.

To address this issue, we introduce a smoothing function to enhance the smoothness of $GIoU_{Loss}$ [49], making the gradient variation smoother when the value of the loss function changes sharply or becomes large; this avoids abrupt fluctuations in the gradients, which is especially effective for handling non-overlapping bounding boxes. The smoothing function defined in this paper is shown in Equation (14):

(14) $$\begin{array}{c} smooth\left(GIoUs\right)=\frac{1}{1-e^{-k\times (GIoUs+1)}} \end{array}$$

where *e* is the natural constant, and *k* represents the constant used to control the degree of smoothing. Notably, smooth(GIoUs) will provide a smooth and gradual transition rather than a sudden change when *x* approaches −1, helping the model to better adapt to the differences between the ground truth box and the predicted box. When dealing with difficult samples, the smooth loss function provides more effective feedback and improves the training performance of the model.

The introduction of the smoothing function resolves the discontinuity in GIoU, which can prevent the sudden changes in the loss function when the value of GIoU approaches −1. Additionally, the smoothing function also alleviates the instability issue of GIoU. The smoothing function ensures that the change in the loss value is smoother when the two bounding boxes are completely non-overlapping and far apart, avoiding the gradient explosion caused by a sharp increase in the loss value. Utilizing smoothing functions to improve $GIoU_{Loss}$ without compromising its original advantages allows it to still effectively quantify the overlap and positional relationship of the bounding boxes while providing a more stable training process in extreme cases where the bounding boxes do not overlap.

**Figure 12:**
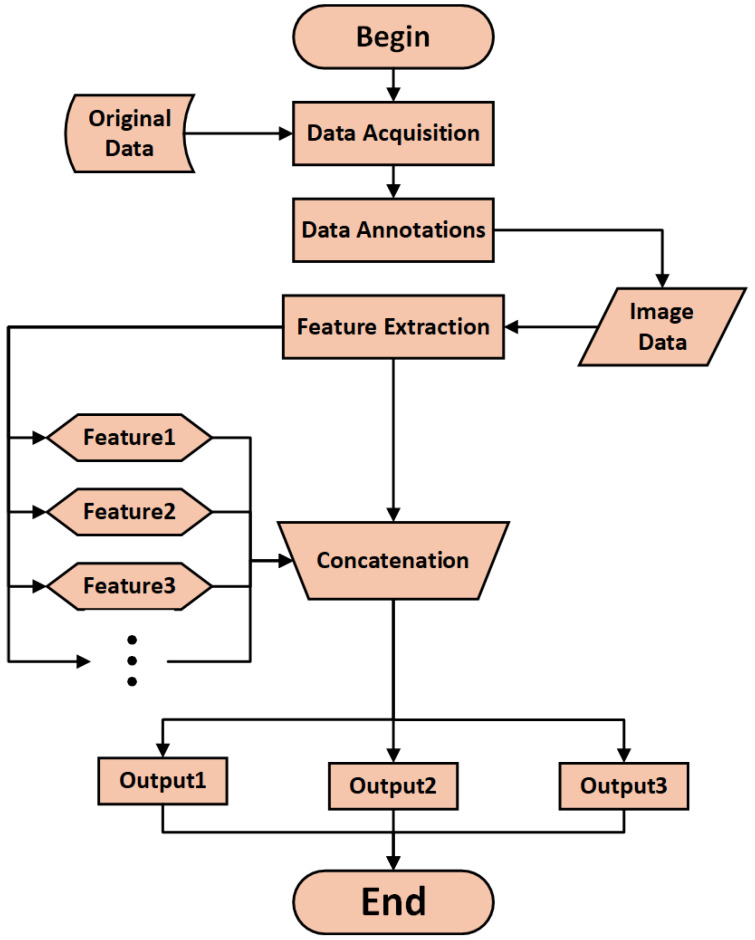
Algorithm flow.

Finally, we have completed our description of the improvements made to YOLOv5, and its algorithm flow is shown in Figure 12.

## 5. Experimental Results

In this section, we first briefly introduce the experimental environment, including the programming language, hardware information, and parameter settings. Then, we introduce the evaluation metrics used in this study, such as accuracy, recall, and mAP. Finally, we analyze the conducted experiments on ADD-dataset and the open-source dataset to verify the effectiveness of DT-YOLO.

### 5.1. The Environment of the Experiment

The setup of the experiments is shown in Table 3. The operating system used was Ubuntu 22.4, which provides a stable platform and strong support for deep learning frameworks. The programming language employed was Python 3.11.9, known for its robust data processing capabilities and rich library ecosystem, making it suitable for machine learning and deep learning experiments. The data processing and deep learning framework primarily depend on NumPy 1.26.3 and PyTorch 2.4.1, enabling efficient matrix operations and deep learning tasks. In addition, we utilized CUDA 12.4 and an NVIDIA GeForce 4090 GPU with 24 GB of memory to accelerate the parallel computation, which is capable of handling large-scale data and complex models of deep learning, significantly improving the efficiency of the experiments. The CPU was an Intel(R) Core(TM) i9-13900K@3.00 GHz processor coupled with 64 GB of RAM, ensuring smooth data processing. The manufacturers of GPU and CPU are NVIDIA and Intel, respectively, and both of them are sourced from Santa Clara, CA, USA.

The key parameters of the training for the model in this study are summarized in Table 4. We set the initial momentum to 0.8 and applied the learning rate warm-up strategy, lasting for three preheating epochs. This ensured that the learning rate gradually increases to its initial value, preventing abrupt changes that could destabilize the optimization process. Then, we adjusted the learning rate dynamically by using a cosine annealing strategy after the formal training had begun.

### 5.2. The Evaluation Metrics

The evaluation metrics used in this study include the typical core metrics for object detection, such as precision, recall, mAP (mean average precision), and GFLOPs (giga floating-point operations per second).

The formulas for precision and recall are shown in Equations (15) and (16), respectively.

(15) $$\begin{array}{c} Precision=\frac{TP}{TP+FP} \end{array}$$

(16) $$\begin{array}{c} Recall=\frac{TP}{TP+FN} \end{array}$$

where TP represents the positive objects that are correctly detected, FP represents the positive objects that are incorrectly detected, and FN represents the negative objects that are incorrectly detected. The precision and recall are inversely related. It may miss FN when the model has strict requirements for precision, reducing the recall. Conversely, it may misclassify some negative objects as positive if the model relaxes the requirements to increase recall, reducing precision.

We propose AP (average precision) for object detection to balance these two metrics and maximize model performance. The model can obtain the precision and recall using different values, which can be plotted on a coordinate axis to generate the P-R (precision-recall) curve. The AP value is the area of the P-R curve. We usually aggregated the arithmetic mean of the AP values across all categories to obtain the mean average precision (mAP) [50], as shown in Equation (17).

(17) $$\begin{array}{c} mAP=\frac{1}{N}\sum_{i=1}^{n}AP_{i}=\frac{1}{N}\sum_{i=1}^{n}\int_{0}^{1}P(R)dR \end{array}$$

where *N* represents the total number of categories in the object detection task, and $AP_{i}$ represents the AP value of the i-th category. We can evaluate the model based on accuracy and recall by effectively using mAP, avoiding the limitation caused by the single metric. During the experiments, different IoU thresholds resulted in different mAP values. To evaluate the model’s performance more accurately, we reached the various mAP values using different IoU thresholds. We averaged them to obtain the final mAP, as shown in Equation (18):

(18) $$\begin{array}{c} mAP_{avg}=\frac{\sum_{i=0.5}^{i=0.95}mAP_{i}}{10},IoU=\left[0.5:0.95:0.05\right] \end{array}$$

where $mAP_{i}$ represents the AP value with the IoU threshold of *i*. The model’s performance can be evaluated more accurately in this way than by using the single IoU threshold. In addition, GFLOPs is also an important evaluation metric in object detection tasks. As a variant of FLOPs, 1 GFLOPs represents performing $10^{9}$ floating-point operations per second. For convolutional layers, the formula is shown in Equation (19):

(19) $$\begin{array}{c} GFLOPs_{conv}=\frac{2\times H^{\prime }\times W^{\prime }\times K\times K\times D\times C}{10^{9}} \end{array}$$

Due to each convolution operation involving one multiplication and one addition, the original basis should be multiplied by 2. For the fully connected layers, the computational complexity of GFLOPs is shown in Equation (20).

(20) $$\begin{array}{c} GFLOPs_{FC}=\frac{2\times N_{in}\times N_{out}}{10^{9}} \end{array}$$

In the object detection task, the total GFLOPs is usually the sum of the computational complexity of all layers due to the convolutional layers and fully connected layers being the core components during the training process, which occupy the main computational resources in the calculation process.

### 5.3. The Comparison Between DT-YOLO and the Baseline Model on ADD-Dataset

We first compared the comprehensive performance of DT-YOLO to the baseline model on ADD-dataset; the evaluation metrics mainly include precision, recall, AP50, AP75, APs, APm, APl, and mAP. The baseline model is the original YOLOv5 model without any changes. The results are shown in Table 5.

**Figure 13:**
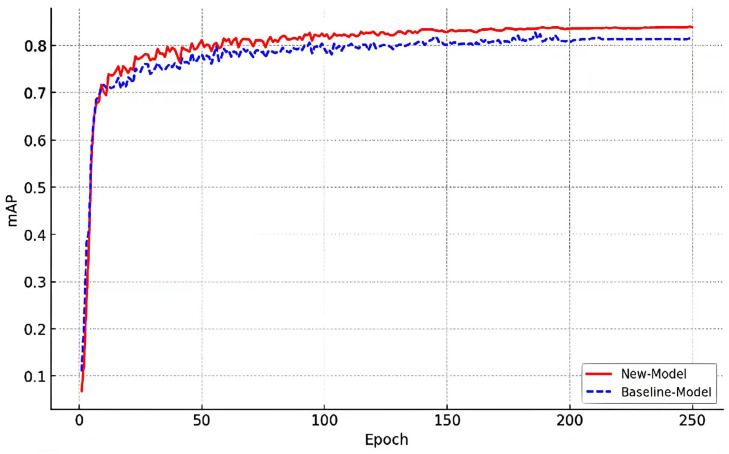
Comparison ofDT-YOLO and the baseline model for mAP@0.5:0.95.

From Table 4, it can be seen that DT-YOLO demonstrates significant improvements across all metrics compared to the baseline model. In detail, DT-YOLO scored 98.5 for precision, which contributes a 3.7 improvement compared to the baseline model, while the recall is 97.6, improving by 3.1 compared to the baseline model. For AP50, DT-YOLO improved by 3.3, reaching 98.4. Furthermore, under the stricter condition of AP75, it also achieved 84.3, improving by 2.9 compared to the baseline model. In addition, DT-YOLO showed a certain degree of improvement in AP values under different conditions, with APs, APm, and APl increasing by 2.7, 2.3, and 2.9, respectively, reaching 75.5, 90.8, and 83.9. Finally, the mAP of DT-YOLO improved by 2.6, reaching 83.9. The results fully demonstrate the accuracy and high reliability of DT-YOLO in the object detection task. The balanced performance in accuracy and recall highlights the exceptional ability of DT-YOLO to correctly identify and minimize missed objects. The improvement across all metrics reflects the strong performance of DT-YOLO in handling increasingly complex and diverse airport apron scenes. To further demonstrate the improvement effect on the performance of DT-YOLO intuitively, we created a comparison curve between DT-YOLO and the baseline model based on the key indicator of mAP, where the IoU threshold was set from 0.5 to 0.95 with a step size of 0.05, as shown in Figure 13.

In Figure 13, the DT-YOLO and baseline models show stable performance growth and eventually converge after iterating for around 250 generations. The proposed DT-YOLO model in this study consistently demonstrated higher mAP levels than YOLO throughout the entire training process, which validates the predictive ability of DT-YOLO in various object detection tasks.

### 5.4. The Results of the Experiments on ADD-Dataset

Comparison and ablation experiments were conducted to evaluate the performance of DT-YOLO comprehensively. The comparison consisted of DT-YOLO being compared with current mainstream object detection models, such as Fast-RCNN, YOLOv3, YOLOv4, YOLOv5, YOLOv6, YOLOv7, YOLOv8, SSD, and RetinaNet. The ablation study removed or added the components to see the impact of each of them.

#### 5.4.1. The Comparison Experiments

To evaluate the performance of each model objectively, we utilized AP50, AP75, APs, APm, APl, mAP, and GFLOPs as the evaluation metrics, where the following applies: AP50 represents the AP value with the IoU threshold set to 0.5; APs, APm, and APl represents the AP value with the IoU threshold set to 0.75; mAP represents the average of the mAPs with various IoU thresholds; and APs, APm, and APl represent the AP values for small, medium, and large objects, respectively. Table 6 indicates the mAP values of the different models, and Table 7 indicates the values of various evaluation metrics in different object detection models.

As shown in Table 6, DT-YOLO performs excellently across all categories in airport apron scenes, achieving the highest AP values compared to other object detection algorithms. In detail, the AP values for the “Engine” and “Nose” categories reached 94.9 and 94.6, respectively, making them the best-performing categories, which indicates that DT-YOLO can effectively and accurately detect these objects. The “tail” category follows closely behind, reaching an AP value of 88.4, demonstrating that although the performance is still relatively strong, it is slightly lower than that of the “engine” and “nose” categories. However, the detection performance for the “Person”, “LandingGear”, and “WingTip” categories is relatively weaker, with AP values of 75.8, 74.8, and 74.9, respectively.

The reason for the results is that the “Engine” and “Nose” categories typically belong to medium- and large-scale objects, which are often located in larger regions of the images, making the objects more prominent and clearer in the images. Additionally, their relatively fixed structure also makes them easier for object detection algorithms to detect. The precise detection of these objects by DT-YOLO can be attributed to its powerful feature extraction and the capability of object localization, which enable DT-YOLO to handle the localization and detection large objects in the complex environments of airport aprons. In contrast, although the “tail” category belongs to medium- and large-scale objects and is generally clear in the images, it presents significant variations in its appearance across different orientations; it even presents as a “straight line” in some extreme scenarios, which leads to some difficulty in detecting “tail” targets accurately. Nonetheless, the detection performance for this category still performs well, highlighting the adaptability and robustness of DT-YOLO in dealing with object appearance variations. On the other hand, for the “Person”, “LandingGear”, and “Wintip” categories, the objects tend to be smaller in size and are significantly affected by various environmental factors, such as shooting angle, distance, and lighting conditions. This can cause these small objects to appear blurred in the image, making it difficult for the algorithm to extract and locate clear features. Furthermore, the small objects are often occluded by complex backgrounds or other objects, further reducing detection accuracy.

Table 7 presents the performance of DT-YOLO compared to other mainstream object detection algorithms on ADD-dataset. From an overall analysis perspective, DT-YOLO outperforms other object detection algorithms in all evaluation metrics, demonstrating its superiority in object detection tasks in complex airport apron scenes. Specifically, DT-YOLO achieves 98.4 and 82.4 for AP50 and AP75, respectively, which indicates that DT-YOLO can accurately detect various types of objects in airport apron scenes. Even under stricter AP75 evaluation criteria, DT-YOLO can still maintain high detection accuracy, proving its superiority and efficiency.

Furthermore, DT-YOLO also performs exceptionally well in detecting objects of different sizes, achieving 75.5, 90.8, and 85.4 for APs, APm, and APl, respectively; these all are superior values to those obtained by the other object detection algorithms. In detail, DT-YOLO excels in detecting medium and large objects, even in complex backgrounds and under varying camera angles, maintaining high accuracy. In contrast, its performance in small-object detection is slightly weaker, as indicated by the previous data analysis. This suggests that small objects may be more prone to detection difficulties due to factors such as blurriness or occlusion. However, DT-YOLO still outperforms other object detection algorithms in detecting small objects and can accurately detect them.

In terms of computational performance, the introduction of the Transformer self-attention mechanism and deformable convolutions increases computation, leading to a slight increase in the value of GFLOPs, reaching 262.9; this is slightly higher than other YOLO-based algorithms by 3 to 6 points. However, the increase remains within an acceptable range, which can meet the real-time detection demands of airport aprons. Additionally, as shown in the table, some of the latest YOLO models, such as YOLOv11, outperform DT-YOLO in a few specific metrics. However, since these models are new and the literature and research on them are limited, there may be potential issues with performance stability. Therefore, considering all factors, DT-YOLO remains superior to the other models. In our future work, we will continue to delve deeper into this area of research.

**Figure 14:**
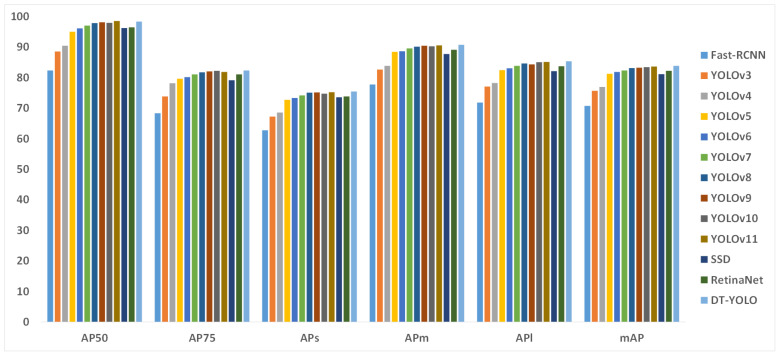
Histogram of different object detection algorithms under various evaluation metrics.

**Figure 15:**
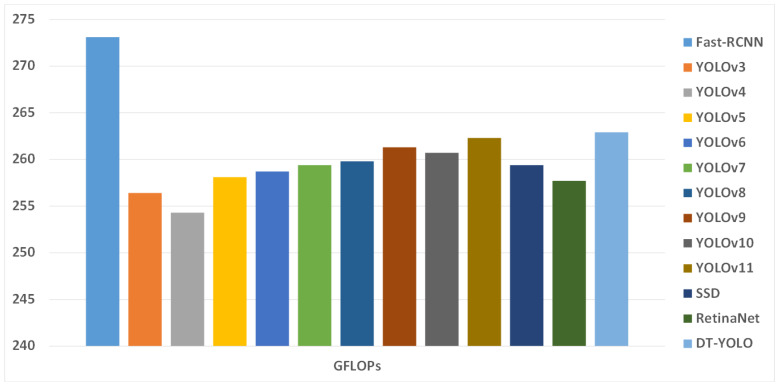
Histogram of GFLOPs.

**Figure 16:**
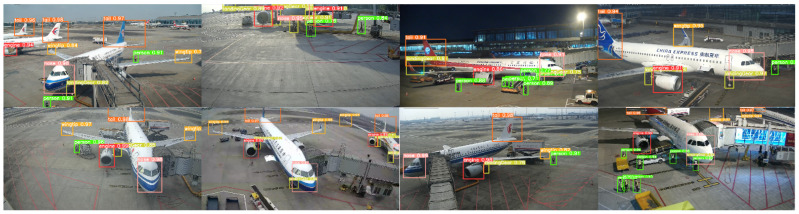
The detection effect of DT-YOLO on ADD-dataset.

In summary, despite the relatively lower detection precision for small objects, DT-YOLO demonstrates a notable performance improvement for all categories compared to other object detection algorithms. This suggests that DT-YOLO not only excels in detecting medium and large objects but also shows potential improvements in detecting small objects in complex and dynamic airport apron environments, proving the superiority and reliability of DT-YOLO. To demonstrate the superiority of DT-YOLO more intuitively, we created a histogram of different object detection algorithms under various evaluation metrics and provided a graph of the detection effect, as shown in Figure 14, Figure 15, Figure 16, respectively.

Figure 13 and Figure 14 visually demonstrate the performance of DT-YOLO in different evaluation metrics and its detection effectiveness. It can be seen from the figures that DT-YOLO outperforms other object detection algorithms in all metrics and can effectively detect key aircraft components and personnel in airport aprons, which indicates that DT-YOLO not only improves accuracy but also successfully shortens the inference time and improves the model’s detection speed. Compared to other models, DT-YOLO demonstrates significant performance advantages. 

#### 5.4.2. The Ablation Experiments

In order to more precisely evaluate the contribution of individual components to the overall performance of the model, we conducted a series of ablation experiments by systematically removing or adding different components of the model. Here, we utilized AP50, AP75, mAP, and GFLOPs as the evaluation metrics. The experiments aimed to provide insights into the contribution of each component, thereby guiding the optimization of the performance for the model, and seven distinct areas of improvement were designed in the ablation experiments. In detail, Improvements 1 to 4 involved adding a single component, including the Transformer self-attention mechanism, the Dropout layer, DCN (deformable convolution network), and the SLoss loss function, respectively. Improvement 5 integrated the D-CTR module, which simultaneously incorporated the Transformer self-attention mechanism and Dropout layer, aiming to enhance the feature representation capabilities of the model. On the basis of integrating the D-CTR module, Improvements 6 and 7 additionally included deformable convolution and the new SLoss loss function, respectively. Each improvement was evaluated through corresponding experiments. The results are shown in Table 8, where “✓” represents the corresponding method.

The experimental results in Table 8 show that adding each component individually in Improvements 1 to 4 does not improve the performance of the model significantly; it even resulted in a decrease in model performance in some cases, which indicates that simply introducing a single component may lead to overfitting or the loss of key feature information, thus negatively affecting overall performance. For example, the Transformer self-attention mechanism in Improvement 1 and the Dropout layer in Improvement 2 caused decreases of 0.6 and 0.8 in the mAP metric, respectively, and also resulted in reductions in the AP50 and AP75 metrics. The reason for these performance drops is that the Transformer self-attention mechanism has strong capabilities in feature extraction and representation, which can cause overfitting. On the other hand, the standalone use of the Dropout layer, while intended to improve generalization, randomly drops some neurons, leading to the loss of some important feature information and a decrease.

In contrast, Improvement 5 integrates the Transformer self-attention mechanism and the Dropout layer into the D-CTR module, which successfully combines the advantages of both components, resulting in a significant improvement in model performance. Compared to the baseline model, Improvement 5 achieved a 1.4 increase in mAP, along with improvements in the AP50 and AP75 metrics. The results indicate that combining the Transformer self-attention mechanism and the Dropout layer can effectively balance the feature extraction of the model and its generalization capabilities, thereby enhancing its overall performance. Improvements 6 and 7 additionally included DCN and the new SLoss loss function on the basis of integrating the D-CTR module, further improving the feature extraction capability of the model. Compared to the baseline model, Improvements 6 and 7 led to increases in mAP of 1.6 and 1.8, respectively, further confirming the positive impact of these components on model performance.

In terms of detection speed, although the addition of new components increased computational complexity, resulting in a slight decrease in GFLOPs, the reduction remained within an acceptable range and met the real-time requirements for airport apron detection tasks. The results demonstrate the effectiveness and applicability of DT-YOLO in dynamic and complex airport apron scenarios.

## 6. Conclusions

In this study, we proposed DT-YOLO (based on YOLOv5) for use in an object detection task regarding the key components of aircraft and staff in airport apron scenes. First, we constructed a dataset named ADD-dataset by randomly capturing images from surveillance videos of airport aprons, including a total of 8643 images, 6 categories, and 80,275 instances, which was sufficient to support the research detailed in this paper. Second, the Transformer self-attention mechanism and a Dropout layer were introduced to construct the D-CTR module, which utilizes the multi-head self-attention mechanism to capture the semantic relationships of distant objects to extract the global dependencies of features, enhancing the expression ability of the model. The Dropout layer helps to mitigate overfitting caused by overly strong feature expressions, improving the model’s generalization ability. Additionally, deformable convolutions were utilized in the feature extraction step to improve the capability of detecting deformed objects in the images, further enhancing the model’s performance. Finally, the GIoULoss function was improved through the introduction of a smoothing function, resulting in a new loss function called SLoss, which addresses the issues of instability and discontinuity in GIoULoss caused by changes in bounding box positions during training. The results of the experiments indicate that DT-YOLO has significantly improved accuracy, when compared to the baseline model and other mainstream object detection models, thus validating the effectiveness of the proposed improvements. Although the addition of the modules decreased the computational efficiency slightly, resulting in a certain increase in GFLOPs, the increase is within an acceptable range in airport apron detection scenarios, meeting the real-time detection needs of airports.

Object detection tasks in airport environments face unprecedented challenges. The introduction of DT-YOLO provides a new approach to address these challenges and outlines future research directions, offering a foundation for the future development of “smart civil aviation”, as well as technological innovation in airports and the civil aviation sector. However, the proposed method still has certain limitations, such as lacking small-object detection abilities. In future research, with the advancements brought by more refined data collection and multi-modal information fusion technologies, we will continue to improve and innovate existing models to achieve greater detection accuracy and efficiency breakthroughs. We will further optimize the structure and application scenarios of DT-YOLO in order to achieve more accurate and efficient object detection.

## Figures and Tables

**Table 1:** Literature Summary.

Paper	Application	Method
[31]	Fault Detection	U-Net for detection of engine blade defects
[32]	Fault Detection	CNN for identifying cracks and burns
[33]	Fault Detection	Replace the YOLOv5 detection module
[34]	Fault Detection	LESM-YOLO for defect detection
[35]	Fault Detection	YOLOv8 for detecting surface defects
[37]	X-ray Detection	EAOD-Net for X-ray detection
[38]	X-ray Detection	X-YOLO for contraband detection
[39]	Remote Sensing	Circular Frequency Filtering algorithm
[40]	Remote Sensing	YOLO-FRS for detecting aircraft
[41]	Remote Sensing	TPH-YOLOv5-Air detecting network

**Table 2:** Information about objects of different scales.

Attributes	Large Objects	Medium Objects	Small Objects
Range	more than 96 × 96 pixels	from 32 × 32 to 96 × 96 pixels	less than 32 × 32 pixels
Number	9312	29,381	41,582
Proportion	11.6%	36.6%	51.8%
Categories	nose, tail, engines	engines, tails	staff, landing gear, wings

**Table 3:** The setup of the experimental environment.

Parameter	Values
Operating System	Ubuntu 22.4
Programming Language	Python 3.11.9
Deep Learning Framework	PyTorch 2.4.1/Cuda 12.4
GPU	NVIDIA GeForce 4090@24 G
CPU	Intel(R) Core(TM) i9-13900K@3.00 GHz
Memory	64 G

**Table 4:** The setup of the training parameter.

Parameter	Value
Initial Learning Rate	0.01
Momentum Factor	0.937
Weight Attenuation Coefficient	0.0005
IoU Training Threshold	0.2
Batch-Size	64
Optimizer	Adma

**Table 5:** Information of objects with different scale.

Model	Precision	Recall	AP50	AP75	APs	APm	APl	mAP
DT-YOLO	98.5	97.6	98.4	84.3	75.5	90.8	85.4	83.9
Baseline	94.8	94.5	95.1	81.4	72.8	88.5	82.5	81.3

**Table 6:** The AP values for each category in different models.

Model	Person	Engine	Nose	LandingGear	WingTip	Tail
Fast-RCNN	63.1	82.9	80.8	60.6	61.3	76.1
YOLOV3	68.3	88.2	86.2	64.2	67.1	80.2
YOLOV4	71.8	80.3	89.4	67.8	69.5	83.1
YOLOV5	74.6	92.0	91.3	71.1	72.0	86.8
YOLOV6	74.9	92.8	92.1	71.7	72.5	87.1
YOLOv7	75.5	93.2	92.8	72.1	73.2	87.8
YOLOv8	75.6	94.1	93.5	73.6	74.1	88.1
YOLOv9	75.7	94.4	93.9	73.9	74.4	87.8
YOLOv10	75.2	93.9	94.2	74.5	73.8	88.2
YOLOv11	75.5	94.6	94.8	74.2	75.1	87.7
SSD	75.2	91.4	90.5	72.6	71.5	85.9
RetinaNet	75.4	93.2	91.9	72.8	73.3	87.1
DT-YOLO	75.8	94.9	94.6	74.8	74.9	88.4

**Table 7:** The Results of Comparison Experiments.

Model	AP50	AP75	APs	APm	APl	mAP	GFLOPs
Fast-RCNN	82.4	68.4	62.8	77.8	71.9	70.8	273.1
YOLOv3	88.6	73.9	67.3	82.7	77.1	75.7	256.4
YOLOv4	90.5	78.2	68.6	83.9	78.3	77.0	254.3
YOLOv5	95.1	79.7	72.8	88.5	82.5	81.3	258.1
YOLOv6	96.2	80.2	73.4	88.7	83.1	81.9	258.7
YOLOv7	97.1	81.1	74.2	89.6	83.9	82.4	259.4
YOLOv8	97.9	81.8	75.1	90.2	84.7	83.4	259.8
YOLOv9	98.2	82.1	75.2	90.5	84.4	83.3	261.3
YOLOv10	98.0	82.3	74.8	90.3	85.1	83.5	260.7
YOLOv11	98.6	81.9	75.3	90.6	85.2	83.7	262.3
SSD	96.3	79.2	73.6	87.8	82.2	81.2	259.4
RetinaNet	96.5	81.1	73.9	89.2	83.8	82.3	257.7
DT-YOLO	98.4	82.4	75.5	90.8	85.4	83.9	262.9

**Table 8:** The Results of Ablation Experiments.

Model	Transformer	Dropout	DCN	SLoss	AP50	AP75	mAP	GFLOPs
Baseline					95.1	79.7	81.3	258.1
Improvement1	✓				94.3	78.6	80.7	256.5
Improvement2		✓			93.9	79.2	80.5	260.2
Improvement3			✓		96.6	79.8	81.7	256.9
Improvement4				✓	96.8	80.5	81.6	257.2
Improvement5	✓	✓			97.5	81.4	82.7	255.3
Improvement6	✓	✓	✓		97.9	81.6	82.9	254.1
Improvement7	✓	✓		✓	97.8	81.7	83.1	253.6
DT-YOLO	✓	✓	✓	✓	98.4	82.4	83.9	252.9

## Data Availability

Data are contained within the article.

## Abbreviations

The following abbreviations are used in this manuscript:

AAD	Airport Apron dataset
DCN	Deformable Convolution Network
CNN	Convolutional Neural Networks
GIOU	Generalized IoU
YOLO	You Only Look Once
TP	True Positives
FP	False Positives
FN	False Negatives
AP	Average Precision
mAP	mean Average Precision
IOU	Intersection Over Union
Adma	Adaptive Moment Estimation
GFLOPs	Giga Floating-point Operations Per Second
